# Speak Forth the Words of Truth and Soberness

**Published:** 1884-07

**Authors:** 


### "SPEAK FORTH THE WORDS OF TRUTH AND SOBERNESS."

[The following extract sufficiently explains itself; it is the glad cry of one who "was blind but now seeth."—ED.]

I say it without fear or favor that yours is the only journal published in the homœopathic profession that stands up manfully and advocates *true Homœopathy*. I have called myself a homœopath and thought I was practicing under the true law. I have bitterly opposed the high-potency theory, calling it "infinitesimal nothingness," and all who advocate or practice with them crazy or idiotic. I have at times in the past dispensed in my practice Schieflin's, McK. & R.'s, P. D. & Co.'s, etc., pills and granules, but, thanks be to your and my esteemed friend, Professor R. R. Gregg, of Buffalo, I am no longer "traveling in darkness," but "light has broken in upon my once clouded vision" and the future seems bright and clear, at least in anticipation. I am satisfied now that the "*true law of cure*" is made known in Hahnemann's *Organon*, and that there is no other. That book, too, should be in the hands of every even pretended follower of Hahnemann. It is, indeed, a medical Bible, and indispensable to him who would practice the healing art in truth and honesty.—R. O. W.

